# Microbiome and Cancers, With Focus on Genitourinary Tumors

**DOI:** 10.3389/fonc.2019.00178

**Published:** 2019-03-26

**Authors:** Alessia Cimadamore, Matteo Santoni, Francesco Massari, Silvia Gasparrini, Liang Cheng, Antonio Lopez-Beltran, Rodolfo Montironi, Marina Scarpelli

**Affiliations:** ^1^Section of Pathological Anatomy, School of Medicine, United Hospitals, Polytechnic University of the Marche Region, Ancona, Italy; ^2^Oncology Unit, Macerata Hospital, Macerata, Italy; ^3^Division of Oncology, S. Orsola-Malpighi Hospital, Bologna, Italy; ^4^Department of Pathology and Laboratory Medicine, Indiana University School of Medicine, Indianapolis, IN, United States; ^5^Department of Pathology and Surgery, Faculty of Medicine, Cordoba, Spain

**Keywords:** microbiome, renal cell carcinoma, immunotherapy, resistance, PD-1 blockade, antibiotic therapy, prostate cancer

## Introduction

Every individual is characterized by a specific “enterotype,” based on the major components of her/his microbiome (i.e., collection of host and microorganism genomes and environmental conditions in an ecosystem) of the gut influenced by diet and geography. This is also influenced by the effects of the organisms present in the infancy as well as the type and pattern of the individual immune system (1).

**Figure 1:**
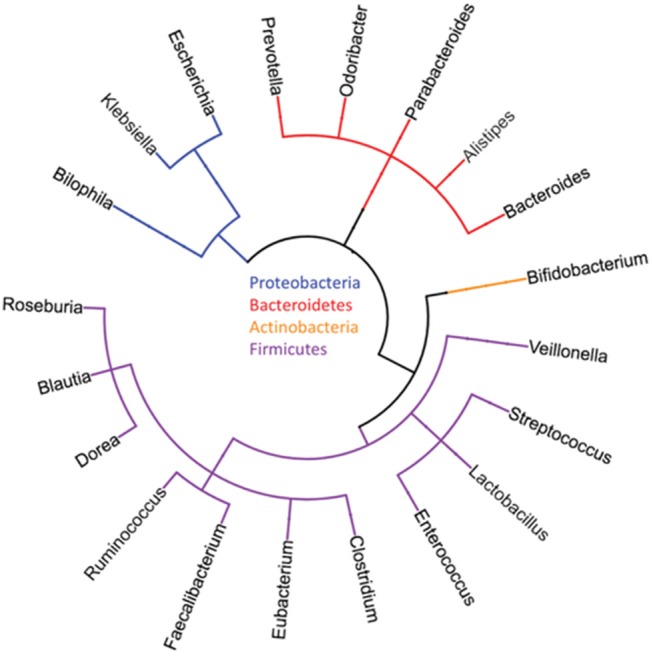
Typical major phyla and genera of the human gut microbiome [reproduced with permission from Goodman and Gardner (4)].

In the last few years, a close biological relationship has emerged among the microbiome of the gut, the metabolism of the body, as well as the immune system including cancer development. With the increasing availability of high-throughput sequencing, single-cell transcriptomics, and mass spectrometry for a very precise characterization of single enteric, neoplastic, and immune cells, and more extensive databases of organisms already sequenced, experimental exploration of this network has become possible. These advances have also included “culturomics” to make an ever-expanding portion of the microbiota investigable, and sophisticated bioinformatics implements in order to achieve data deconvolution and combination. In 2008, the Human Microbiome Project (HMP) started to characterize the microbial communities from 300 healthy individuals, providing one of the broadest microbial genome databases targeting different body sites: nares, oral cavity, skin, gastrointestinal tract, breast, and urogenital tract (2, 3) (Figure 1).

## Microbiome and Cancer

In 2017, many experimental studies had been published to demonstrate the importance of single bacterial species on the intestine, the individual immune response and cancer progression, and response to therapy. One of the first unexpected pieces of evidence was that secondary tumor deposits in patients with colorectal cancers include bacteria, such as Fusobacterium species, including Bacteroides, Prevotella, and Selenomonas species, and its associated microbiome. These findings demonstrated the microbiome stability between paired primary and metastatic tumors. Antibiotic treatment of Fusobacterium-positive colon cancers mice-xenografts reduces tumor growth, cancer cell proliferation along with Fusobacterium load, which favors the hypothesis that Fusobacterium species is associated with neoplastic progression (5, 6).

In breast cancer, bacterium Methylobacterium radiotolerans was found relatively enriched compared to normal adjacent tissue from the same patient. Furthermore, bacterial DNA load was reduced in cancer samples vs. healthy tissue and correlated inversely with advanced disease (7, 8). In the distal esophagus, the impact of the microbiome in the pathogenesis of reflux-related disorders and in the development of intestinal metaplasia is well-demonstrated. Patients with esophagitis and Barrett's esophagus have a greater proportion of gram-negative anaerobes/microaerophiles with respect the normal controls. This altered microbiome may promote Barrett's metaplasia and progression to adenocarcinoma (9, 10). The compositions of bacteria community and the throat biodiversity in laryngeal carcinoma patients compared to a control population were different and might be a risk factor for laryngeal carcinoma (11).

The most clinical-affecting evidence regarding cancer microbiome is its contribution to therapy resistance. In pancreatic cancer the most common species identified belong to the Enterobacteriaceae and Pseudomonadaceae families. Enterobacteriaceae express a bacterial enzyme cytidine deaminase (CDD) isoform that confer resistance to gemcitabine. Supporting this, co-treatment with the antibiotic ciprofloxacin abrogate the gemcitabine resistance in colon cancer mouse models (12).

On the other side, there is evidence that corroborates the hypothesis of a protection role of microbiome toward neoplastic changes. Hence, results show that individuals with microbiota linked to a plant diet are the ones with a lower incidence of cancer of the colon (13). Such a diet stimulates bacteria to produce short-chain fatty acids (SCFAs), particularly butyrate, propionate, and acetate. These fatty acids show an anti-inflammatory property through the induction of T-regulatory cells of colonic tissues. Connections of microbiome, production of short-chain fatty acids, and the immune system become more interesting when researchers started to explore the influence of the microbiome in relation to immunotherapy drugs response.

## Microbiome and Immunotherapy Drugs Response

Immunotherapy based on and programmed death-ligand 1 (PD-L1)- and programmed death 1 (PD-1)-targeted antibodies has profoundly modified the prognostic and therapeutic landscape for many types of tumors, with demonstrated efficacy against renal cell carcinoma (RCC), non–small cell lung cancer (NSCLC), and melanoma. PD-L1 tissue expression is a poor prognostic factor as well as a predictor of good responses from both PD-1 and PD-L1 inhibitors in urothelial carcinoma (UC) and RCC (14). In a recent meta-analysis on the expression of PD-1 and PD-L1 in solid tumors, as a predictive biomarker of benefit from PD-1/PD-L1 axis inhibitors, odds ratios of objective response in PD-L1–positive patients compared with PD-L1–negative patients was 2.34 for RCC and 2.20 for bladder cancer (15). Liu et al. also confirmed that “patients with higher ratios of PD-L1-positive cells responded significantly better to both PD-1 and PD-L1 antibodies than those with lower ratios of PD-L1-positive cells” (16).

Each PD-1/PD-L1 drug approved by FDA is associated with a PD-L1, a immunohistochemistry (IHC)-based tissue assay. IHC-based PD-L1 assay is basically utilized to potentially predict the response to anti-PD-1 or/and anti-PD-L1 therapies. A fraction of patients with a negative IHC assay can show a response. This means that identification and utilization of other biomarkers is of great importance for a better selection of patients who might respond to such therapies.

Primary resistance to Immune checkpoint inhibitors (ICIs) has been linked to different factors, including poor intrinsic antigenicity of malignant cells, lack of priming by potentially immunogenic pretreatment with radio-, or/and chemotherapy (17), poor antigen presentation at the time of the priming phase (18), immunosuppression exerted locally by extracellular metabolites (19), and functional exhaustion of lymphocytes infiltrating the tumor (20, 21). On the contrary, high mutational burden and high immunogenic antigenicity of malignant cells are in favor of a better response to ICIs (22, 23). Recently, Routy et al. demonstrated that abnormal gut microbiome composition could have an influence on primary resistance to PD-1blockade in mice xenografts and patients with cancer. In particular, they showed that the clinical benefit of ICIs in patients with cancer at an advanced stage is inhibited by antibiotic therapy (ATB) (24). They tested the effect of ATB on patients with advanced UC, RCC, or NSCLC, who had received PD-1/PD-L1mAb following one or several previous therapies. Progression-free survival (PFS) and overall survival (OS) were significantly shorter in the ATB-treated cohort when either all patients were combined together or when individual cancer types were investigated. In univariate and multivariate analyses, ATB was a predictor factor for resistance to PD-1 blockade, not dependent from traditional prognostic markers in RCC and NSCLC. To evaluate the composition of the microbiota of the gut, they used quantitative metagenomics with analysis of the data in a reference catalog of 9.9 million genes. The greatest richness of the samples, analyzed at the levels of metagenomic species (MGS) and gene count, was correlated with the clinical response. This was defined by the lack of progression of disease 6 months following the initiation of ICIs.

*Akkermansia muciniphila* (A. muciniphila) was the commensal associated most significantly with excellent clinical outcomes in both RCC and NSCLC. When analyzing memory T cell responses from the peripheral blood of the patients, stimulated against microbiota following initiation of PD-1 blockade, the only immune response linked with the clinical benefit at the time of immunotherapy was the Th1 and Tc1 cell reactivity against A. muciniphila. This immunomodulatory effect might be explained by the production of SCFAs, such as propionate and acetate, by A. muciniphila. These short-chain fatty acids are ligands of the two orphan G-protein–coupled receptors 41 and 43 (GPR41 and GPR43). The former regulates the tumor cells apoptosis induced by SCFA and so exerts a tumor suppressor activity. Furthermore, propionate produced by the bacterium inhibits histone deacetylases and thus increases the histone hyperacetylation.

**Figure 2:**
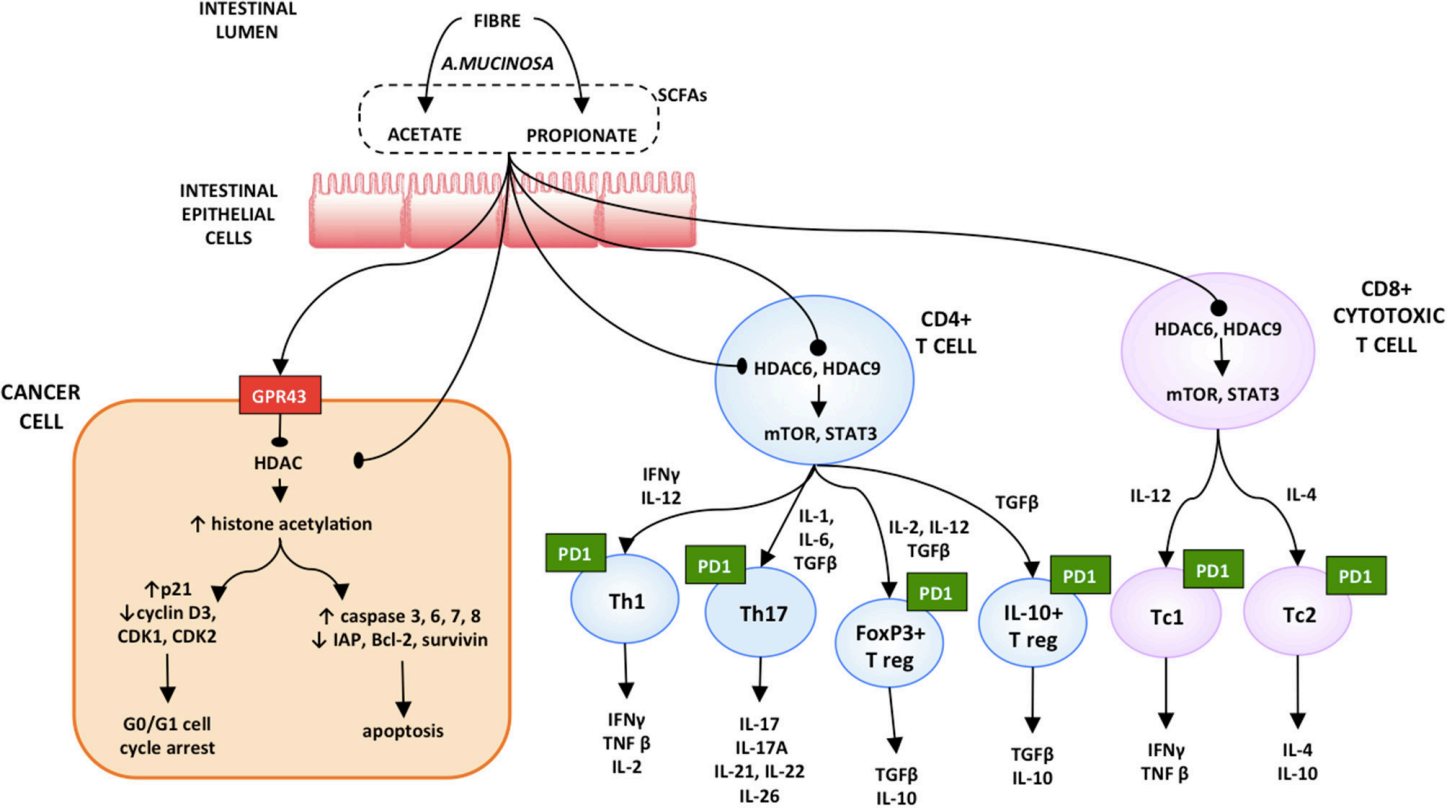
Proposed model on the role of microbiota in resuming the response to immune checkpoint inhibitors [reproduced with permission from Santoni et al. (28)].

The inhibition of the expression of Histone Deacetylases (HDACs) has several effects, ranging from a pro-apoptotic activity to a pro-inflammatory response. By opening cell chromatin and thus increasing the DNA accessibility to transcription factors, the histone hyperacetylation induces overexpression of caspases 6, 7, and 8, including caspase 3, and reduces the inhibitor of apoptosis (IAP) family expression (25). The inhibition of expression the HDACs activates the mTOR-S6K and STAT3 pathways. All this stimulates Th17, Th1, FoxP3+, and IL-10+ T cells, as well as the production of IL-10, IFN-g, and IL-17 in CD8+ T cells in both Tc1- and Tc17-cell subsets (26). Moreover, propionate promotes T-cell migration by increasing the expression of intercellular adhesion molecule 1 (ICAM-1) and E-selectin on endothelial cells (27, 28) (Figure 2).

Derosa et al. demonstrated the effect of ATB in patients with RCC and NSCLC treated with anti-PDL1 mAb monotherapy or combination therapy. In patients with RCC, ATB compared with no ATB was linked with an increased risk of progressive disease, shorter PFS, and shorter OS. Similar rates were also obtained in the NSCLC cohort (29). Researchers are now planning to transfer fecal bacteria from patients who respond to treatment with checkpoint inhibitors into the intestine of non-responder patients. This process is currently called “fecal microbiome transplant.” Microbiota composition might also be manipulated by the application of foods and prebiotics. The prevalence of a subspecies selected by diet rather than others could modify the population predisposition to a specific disease and the response to therapy of cancer patients (30). Fecal microbiota transplant, although not being probiotic, could be considered a fermented food, given the microbes, and nutrients present.

## Microbiome in Bladder and Prostate Cancer

Of great interest is the urinary microbiota profile investigated by Wu et al. They analyzed DNA from urine pellet collected from male patients with urothelial carcinoma and non-neoplastic controls. They observed enrichment of some bacterial genera (such as Sphingobacterium, Anaerococcus, and Acinetobacter) and decrease of others (such as Roseomonas, Proteus, and Serratia) in the group with cancer in comparison with the control group. Patients with high risk of recurrence and progression had an enrichment of Herbaspirillum, Porphyrobacter, and Bacteroides. This means these bacteria can be considered as potential biomarkers in risk stratification (31).

In the last year, interesting results have emerged by investigations on the microbiome in PCa patients. The microflora of tumor, peri-tumor, and benign prostate tissue samples have recently been characterized by massive ultradeep pyrosequencing. Interestingly, differences in microbial populations among paired tumor/peri-tumor and non-tumor prostate tissues have been detected. This finding generates the hypothesis that the distribution of bacterial microbes varies according to the nature of tissue within the same gland. This suggests a pathophysiological association between the local microbial niche and composition, and the tumor itself (32, 33) (Table 1).

**Table 1:** Metagenomic human studies identifying microbiota associated with cancer tissues [reproduced with permission from Goodman and Gardner (4)].

**Tissue type**	**Species differential**	**References**
Colorectal cancer	*Fusobacterium, Selenomonas*, and *Leptotrichia* species increased in cancer tissues	(5, 6)
Breast cancer	*Alistipes, Sphingomonas*, and *Methylbacterium* increased in cancer tissue	(7, 8)
Esophageal cancer	*Streptococcus, Prevotella*, and *Veillonella* species increased in cancer tissues	(9, 10)
Head and neck cancer	*Fusobacterium, Prevotella*, and *Gemella* species increased in cancer tissues; *Streptococcus* and *Rothia* species decreased in cancer tissues	(11)
Pancreatic cancer	Enterobacteriaceae, Pseudomonadaceae, Moraxellaceae, and Enterococcaceae increased in cancer tissues	(12)
Prostate cancer	*Propionobacterium acnes* increased in cancer tissues	(32–37)

A case-control pilot study has been conducted by Golombos et al. to demonstrate the impact of the gut microbiota on PCa pathogenesis. They performed a computational genomics analysis on stool samples of men with benign prostatic conditions and men with intermediate or high risk clinically localized PCa. Biologically significant abundance differences of bacteria species and 23 metabolic differentially abundant pathways were identified between the two cohorts (34). Likewise, analyses on the urinary microbiome showed a prevalence of uropathogens and pro-inflammatory bacteria differentially abundant in PCa patients compared to healthy subjects in urine collected from men prior to biopsy for PCa (35).

Liss et al. developed a microbiome-derived risk profile for PCa, derived from altered metabolic pathways, comparing the taxonomic composition of samples (64 with PCa and 41 without) of rectal swab collected 2 weeks before prostate biopsy (36). Even though the differences between the two groups are not impressive, these results are hypothesis-generating and pave the way to further evaluate the manipulation of aberrant microbiomes to reduce PCa risk (3).

The composition of the microbiota in the gut is influenced by oral androgen receptor axis-targeted therapies (ATT) in prostate cancer patients. Results on fecal microbiota profile shows the abundance of species linked to response to anti-PD-1 immunotherapy, including Ruminococcaceae spp., and A. muciniphila, and an greater representation of bacterial gene pathways that are involved in steroid biosynthesis as well as steroid hormone biosynthesis in the fecal microbiota of men under ATT (4, 37). Additional studies are needed to evaluate whether the gut microbiota can influence clinical responses to ATT, and modulate the anticancer effects of future therapies, including immunotherapy.

As regards to genitourinary tumors, there are only few trials ongoing (38, 39). One to take into consideration is a prospective study on prostate cancer and breast cancer patients who are undergoing two different standards of care radiation regimens. Exposure to radiation can impact immune cells that are present in the blood as well as the underlying microbiota. The aim of this study is to study microbial changes and how these changes correlate with alteration in immune mediators (i.e., lymphocytes, cytokines) present in blood samples before, during, and after radiation, by collecting stool specimens at baseline, end of radiation therapy and during the follow up (ClinicalTrials.gov Identifier: NCT03383107).

## Conclusions

The host and the microbiota share a complex balanced relationship that can be overthrown in a state of dysbiosis consequential to environmental changes. Alteration of this balance could lead to promotion of inflammatory diseases and cancer. There is evidence showing that the activity of microbiota in the restoration of response to immune checkpoint inhibitors involves both the immune and cancer cells. Stimulating recall Th1 responses against A. muciniphila improves immunosurveillance in cancer patients. Microbiome composition has the potential to be a novel biomarker of response to ICIs and a therapeutic opportunity for unresponsive patients. In patients with RCC, antibiotic therapy was linked to an increased risk of progressive disease, shorter PFS, and shorter OS. Pioneer studies on bladder and prostate cancer patients' microbiome pave the way to the investigation of a possible novel prognostic, diagnostic, and therapeutic tool.

## Author Contributions

RM and MSc conception and design. AC drafting the manuscript. FM, MSa and SG review of the literature. LC and AL-B critical revision of the manuscript.

### Conflict of Interest Statement

The authors declare that the research was conducted in the absence of any commercial or financial relationships that could be construed as a potential conflict of interest.

## References

[1] Arumugam M Raes J Pelletier E Le Paslier D Yamada T Mende DR. Enterotypes of the human gut microbiome. Nature. (2011) 473:174–80. 10.1038/nature09944 21508958 PMC3728647

[2] Lloyd-Price J Mahurkar A Rahnavard G Crabtree J Orvis J Hall AB. Strains, functions and dynamics in the expanded human microbiome project. Nature. (2017) 550:61–6. 10.1038/nature23889 28953883 PMC5831082

[3] Cavarretta I Mancini N Salonia A. Analysis of the enteric microbiome: first tentative steps towards a comprehensive work-up of prostate cancer? Eur Urol. (2018). 74:583–584. 10.1016/j.eururo.2018.07.009 30037526

[4] Goodman B Gardner H. The microbiome and cancer. J Pathol. (2018) 244:667–76. 10.1002/path.5047 29377130

[5] Bullman S Pedamallu CS Sicinska E Clancy TE Zhang X Cai D. Analysis of Fusobacterium persistence and antibiotic response in colorectal cancer. Science. (2017) 358:1443–8. 10.1126/science.aal5240 29170280 PMC5823247

[6] Kostic AD Gevers D Pedamallu CS Michaud M Duke F Earl AM. Genomic analysis identifies association of Fusobacterium with colorectal carcinoma. Genome Res. (2012) 22:292–8. 10.1101/gr.126573.111 22009990 PMC3266036

[7] Xuan C Shamonki JM Chung A Dinome ML Chung M Sieling PA. Microbial dysbiosis is associated with human breast cancer. PLoS ONE. (2014) 9:e83744. 10.1371/journal.pone.0083744 24421902 PMC3885448

[8] Chan AA Bashir M Rivas MN Duvall K Sieling PA Pieber TR. Characterization of the microbiome of nipple aspirate fluid of breast cancer survivors. Sci Rep. (2016) 6:28061. 10.1038/srep28061 27324944 PMC4914981

[9] Yang L Lu X Nossa CW Francois F Peek RM Pei Z. Inflammation and intestinal metaplasia of the distal esophagus are associated with alterations in the microbiome. Gastroenterology. (2009) 137:588–97. 10.1053/j.gastro.2009.04.046 19394334 PMC2963147

[10] Snider EJ Freedberg DE Abrams JA. Potential role of the microbiome in barrett's esophagus and esophageal adenocarcinoma. Dig Dis Sci. (2016) 61:2217–25. 10.1007/s10620-016-4155-9 27068172 PMC4945493

[11] Gong HL Shi Y Zhou L Wu CP Cao PY Tao L. The composition of microbiome in larynx and the throat biodiversity between laryngeal squamous cell carcinoma patients and control population. PLoS ONE. (2013) 8:e66476. 10.1371/journal.pone.0066476 23824228 PMC3688906

[12] Geller LT Barzily-Rokni M Danino T Jonas OH Shental N Nejman D. Potential role of intratumor bacteria in mediating tumor resistance to the chemotherapeutic drug gemcitabine. Science. (2017) 357:1156–60. 10.1126/science.aah5043 28912244 PMC5727343

[13] Smith PM Howitt MR Panikov N Michaud M Gallini CA Bohlooly-Y M. The microbial metabolites, short-chain fatty acids, regulate colonic Treg cell homeostasis. Science. (2013) 341:569–73. 10.1126/science.1241165 23828891 PMC3807819

[14] Mann SA Lopez-Beltran A Massari F Pili R Fiorentino M Koch MO. Targeting the programmed cell death-1 pathway in genitourinary tumors: current progress and future perspectives. Curr Drug Metab. (2017) 18:700–11. 10.2174/1389200218666170518162500 28524003

[15] Khunger M Hernandez AV Pasupuleti V Rakshit S Pennell N Stevenson JG Programmed cell death 1 (PD-1) ligand (PD-L1) expression in solid tumors as a predictive biomarker of benefit from PD-1/PD-L1 axis inhibitors: a systematic review and meta-analysis. JCO Precision Oncol. (2017) 1:1, 1–15. 10.1200/PO.16.00030 35172490

[16] Liu J Zhang C Hu J Tian Q Wang X Gu H. Effectiveness of anti-PD-1/PD-L1 antibodies in urothelial carcinoma patients with different PD-L1 expression levels: a meta-analysis. Oncotarget. (2018) 9:12400–7. 10.18632/oncotarget.24249 29552320 PMC5844756

[17] Carbone DP Reck M Paz-Ares L First-line nivolumab in stage iv or recurrent non-small-cell lung cancer. N Engl J Med. (2017) 376:2415–26. 10.1056/NEJMoa1613493 28636851 PMC6487310

[18] Schumacher TN Schreiber RD. Neoantigens in cancer immunotherapy. Science. (2015) 348:69–74. 10.1126/science.aaa4971 25838375

[19] Spranger S Bao R Gajewski TF. Melanoma-intrinsic β-catenin signalling prevents anti-tumour immunity. Nature. (2015) 523:231–5. 10.1038/nature14404 25970248

[20] Smyth MJ Ngiow SF Ribas A Teng MW. Combination cancer immunotherapies tailored to the tumour microenvironment. Nat Rev Clin Oncol. (2016) 13:143–58. 10.1038/nrclinonc.2015.209 26598942

[21] Koyama S Akbay EA Li YY Herter-Sprie GS Buczkowski KA Richards WG. Adaptive resistance to therapeutic PD-1 blockade is associated with upregulation of alternative immune checkpoints. Nat Commun. (2016) 7:10501. 10.1038/ncomms10501 26883990 PMC4757784

[22] Rizvi NA Hellmann MD Snyder A. Cancer immunology. Mutational landscape determines sensitivity to PD-1 blockade in non-small cell lung cancer. Science. (2015) 348:124–8. 10.1126/science.aaa1348 25765070 PMC4993154

[23] Riaz N Havel JJ Kendall SM Makarov V Walsh LA Desrichard A. Recurrent SERPINB3 and SERPINB4 mutations in patients that respond to Anti-CTLA4 immunotherapy. Nat Genet. (2016) 48:1327–9. 10.1038/ng.3677 27668655 PMC5553281

[24] Routy B Le Chatelier E Derosa L Duong CPM Alou MT Daillère R. Gut microbiome influences efficacy of PD-1-based immunotherapy against epithelial tumors. Science. (2018) 359:91–7. 10.1126/science.aan3706 29097494

[25] Tang Y Chen Y Jiang H Robbins GT Nie D. G-protein-coupled receptor for short-chain fatty acids suppresses colon cancer. Int J Cancer. (2011) 128:847–56. 10.1002/ijc.25638 20979106

[26] Park J Kim M Kang SG Jannasch AH Cooper B Patterson J Short-chain fatty acids induce both effector and regulatory T cells by suppression of histone deacety-lases and regulation of the mTOR-S6K pathway. Mucosal Immunol. (2015) 8:80–93. 10.1038/mi.2014.44 24917457 PMC4263689

[27] Miller SJ Zaloga GP Hoggatt AM Labarrere C Faulk WP. Short-chain fatty acids modulate gene expression for vascular endothelial cell adhesion molecules. Nutrition. (2005) 21:740–8. 10.1016/j.nut.2004.11.011 15925300

[28] Santoni M Piva F Conti A Santoni A Cimadamore A Scarpelli M. Re: Gut microbiome influences efficacy of PD-1-based immunotherapy against Epithelial Tumors. Eur Urol. (2018) 74:521–2. 10.1016/j.eururo.2018.05.033 29891391

[29] Derosa L Hellmann MD Spaziano M Halpenny D Fidelle M Rizvi H. Negative association of antibiotics on clinical activity of immune checkpoint inhibitors in patients with advanced renal cell and non-small-cell lung cancer. Ann Oncol. (2018) 29:1437–44. 10.1093/annonc/mdy103 29617710 PMC6354674

[30] Reid G Microbes in food to treat and prevent disease. Exp Rev Precision Med Drug Dev. (2018) 3:79–81. 10.1080/23808993.2018.1429217

[31] Wu P Zhang G Zhao J Chen J Chen Y Huang W Profiling the urinary microbiota in male patients with bladder cancer in China. Front Cell Infect Microbiol. (2018) 8:167 10.3389/fcimb.2018.00167 29904624 PMC5990618

[32] Cavarretta I Ferrarese R Cazzaniga W Saita D Lucianò R Ceresola ER. The microbiome of the prostate tumor microenvironment. Eur Urol. (2017) 72:625–31. 10.1016/j.eururo.2017.03.029 28434677

[33] Fassi Fehri L Mak TN Laube B Brinkmann V Ogilvie LA Mollenkopf H. Prevalence of Propionibacterium acnes in diseased prostates and its inflammatory and transforming activity on prostate epithelial cells. Int J Med Microbiol. (2011) 301:69–78. 10.1016/j.ijmm.2010.08.014 20943438

[34] Golombos DM Ayangbesan A O'Malley P Lewicki P Barlow L Barbieri CE. The role of gut microbiome in the pathogenesis of prostate cancer: a prospective, pilot study. Urology. (2018) 111:122–8. 10.1016/j.urology.2017.08.039 28888753

[35] Shrestha E White JR Yu SH Kulac I Ertunc O De Marzo AM. Profiling the urinary microbiome in men with positive versus negative biopsies for prostate cancer. J Urol. (2018) 199:161–71. 10.1016/j.juro.2017.08.001 28797714 PMC5937117

[36] Liss MA White JR Goros M Gelfond J Leach R Johnson-Pais T. Metabolic biosynthesis pathways identified from fecal microbiome associated with prostate cancer. Eur Urol. (2018) 74:575–82. 10.1016/j.eururo.2018.06.033 30007819 PMC6716160

[37] Sfanos KS Sauvageot J Fedor HL Dick JD De Marzo AM Isaacs WB. A molecular analysis of prokaryotic and viral DNA sequences in prostate tissue from patients with prostate cancer indicates the presence of multiple and diverse microorganisms. Prostate. (2008) 68:306–20. 10.1002/pros.20680 18163428

[38] Markowski MC Boorjian SA Burton JP Hahn NM Ingersoll MA Maleki Vareki S. The microbiome and genitourinary cancer: a collaborative review. Eur Urol. (2019). 75, 637–646. 10.1016/j.eururo.2018.12.043 30655087 PMC9774685

[39] Sfanos KS Markowski MC Peiffer LB Ernst SE White JR Pienta KJ. Compositional differences in gastrointestinal microbiota in prostate cancer patients treated with androgen axis-targeted therapies. Prostate Cancer Prostatic Dis. (2018) 21:539–48. 10.1038/s41391-018-0061-x 29988102 PMC6283851

